# Species variations in the gut microbiota of captive snub-nosed monkeys

**DOI:** 10.3389/fendo.2023.1250865

**Published:** 2023-09-13

**Authors:** Li Xi, Jincheng Han, Xiaohui Wen, Longfei Zhao, Xinxi Qin, Shengjun Luo, Dianhong Lv, Shuai Song

**Affiliations:** ^1^ College of Biology and Food, Shangqiu Normal University, Shangqiu, China; ^2^ Henan Engineering Research Center of Development and Application of Green Feed Additives, College of Biology and Food, Shangqiu Normal University, Shangqiu, China; ^3^ Institute of Animal Health, Guangdong Academy of Agricultural Sciences, Guangzhou, China; ^4^ College of Veterinary Medicine, Northwest A&F University, Yangling, China

**Keywords:** snub-nosed monkey, gut microbiota, captivity, species, conservation

## Abstract

**Introduction:**

Snub-nosed monkeys are species in danger of extinction due to habitat fragmentation and human activities. Captivity has been suggested as an Auxiliary Conservation Area (ASA) strategy. However, little is known about the adaptation of different species of snub-nosed monkeys to captive environments.

**Methods:**

This study compared the gut microbiota between *Rhinopithecus bieti*, *R. brelichi*, and *R. roxellana* under identical captive conditions to provide insights for improving captive conservation strategies.

**Results:**

The results showed that these three *Rhinopithecus* species shared 80.94% of their Operational Taxonomic Unit (OTU), indicating high similarity in gut microbiota composition. The predominant phyla were Firmicutes and Bacteroidetes for all three *Rhinopithecus* species, but differences were observed in diversity, characteristic bacterial communities, and predicted function. Significant enrichment of cellulolytic families, including Ruminococcaceae, Clostridiales vadinBB60 group, Christensenellaceae, and Erysipelotrichaceae, and pathways involved in propionate and butyrate metabolism in the gut of *R. bieti* suggested that it may have a superior dietary fiber utilization capacity. In contrast, Bacteroidetes, Ruminoccaceae, and Trichospiraceae were more abundant in *R. brelichi* and *R. roxellana*, and were associated with saccharide and glycan metabolic pathways. Moreover, *R. brelichi* and *R. roxellana* also had higher similarity in microbiota composition and predicted function.

**Discussion:**

In conclusion, the results demonstrate that host species are associated with the composition and function of the gut microbiota in snub-nosed monkeys. Thus, host species should be considered when formulating nutritional strategies and disease surveillance in captive snub-nosed monkeys.

## Introduction

Hosts and gut microbiota rely on each other and maintain a dynamic balance through long-term mutual adaptation, forming a mutualistic relationship. This dynamic equilibrium is important for maintaining host health and metabolic stability, particularly the intestinal environment’s homeostasis (1). Gut microbes can provide substrates and enzymes for host metabolism, help digest nutrients in food, synthesize vitamins, and regulate energy metabolism (2, 3). The host’s physiological structure and immune system largely regulate and restrain the colonization of gut microbes, and the gut microbiota composition changes based on the host’s diet, physiological state, and external environment (4).

Snub-nosed monkeys belong to *Rhinopithecus* in the Primate, Cercopithecidae and Colobinae, which includes five endangered species: *R. bieti*, *R. brelichi*, *R. roxellana*, *R. strykeri*, and *R. avunculu*. Zhou et al. (2014) found that the northern species (*R. brelichi* and *R. roxellana*) and the Himalayan species (*R. bieti* and *R. strykeri*) diverged about 1.6 million years ago (5). Snub-nosed monkeys are typical phytophagous animals that mainly feed on shoots, young buds, fresh leaves, mosses and fruits. Snub-nosed monkeys have a multicapsular stomach, and the microbes that live in the stomach can promote the digestion of plant fibers (5). In addition, the hindgut of snub-nosed monkeys is also dominated by bacteria capable of producing complex carbohydrate-degrading enzymes (6), which helps snub-nosed monkeys to maintain physiological functions and consistently gain energy from high-fiber foods during the long winters and low temperatures (7).

Habitat variations often trigger adaptive changes in the gut microbiota of snub-nosed monkeys. Hale et al. (2019) found that the richness of gut microbiota and the population of cellulolytic bacteria (Lachnospiraceae and Ruminococcaceae) decreased significantly in captive *R. brelichi* compared to their wild counterparts, while *Prevotella* and *Bacteroides*, which can break down simple sugars and carbohydrates, increased significantly to adapt to the captive diet (8). Wang et al. (2021) similarly observed lower diversity and richness of gut microbiota in captive *R. roxellana* compared to the wild ones, accompanied by a significant increase in *Prevotella*/*Bacteroides* ratio, suggesting an enhanced ability to digest simple sugars (9). Even when their habitat remains unchanged, artificial feeding still affects the gut microbiota of snub-nosed monkeys. Feeding peanuts, apples, and carrots to wild *R. roxellana* and *R. bieti* significantly reduced Firmicutes population and increased Bacteroidetes population (10, 11). Therefore, while captivity or artificial assisted feeding can be important for the conservation of snub-nosed monkeys, changes in habitat and food structure can affect their physiology and gut health. *R. bieti*, *R. brelichi*, and *R. roxellana* are only distributed in the mountainous areas of southwest China. Investigating the adaptation of these three *Rhinopithecus* species to captive environments can facilitate the development of conservation strategies for them. In this study, Illumina NovaSeq high-throughput sequencing was used to identify the characteristics of gut microbiota in *R. bieti*, *R. brelichi*, and *R. roxellana* that were captivated in the Beijing Zoo. By comparing similarities and differences in their microbial composition and predicted functions, this research provides a reference for disease surveillance and dietary adjustment during the conservation of captive snub-nosed monkeys.

## Materials and methods

### Sample collection

Fresh feces were collected from male *R. bieti*, *R. brelichi*, and *R. roxellana*, aged between 2-9 years old, with a sample size of 5 for each species, in the Beijing Zoo in February 2023. The snub-nosed monkeys were kept in three adjacent enclosures and provided with a uniform diet including fresh leaves, fruits, vegetables, cooked corn cakes, eggs, and beef strips, etc. Three months prior to stool collection, no snub-nosed monkeys had a history of disease or medication.

### High-throughput sequencing of the fecal microbiota

The total DNA of fecal microbiota was extracted using the GenElute™ Stool DNA Isolation Kit (Sigma-Aldrich, USA) according to the instructions. DNA quality was detected using a Nanodrop 2000 ultra-trace spectrophotometer (Thermo Scientific, USA) and 1% agarose gel electrophoresis. The V3-V4 region of the bacterial 16S rRNA gene was amplified using universal primers (338F: 5’-ACTCCTACGGGAGGCAGCAG-3’ and 806R: 5’-GGACTACHVGGGTWTCTAAT-3’) and three repeats for each sample. The PCR was performed in a total reaction volume of 10 μL: template DNA 10 ng, 2.0 mmol/L dNTPs 2 μL, KOD FX Neo Buffer 5 μL, KOD FX Neo 0.2 μL, 10 μmol/L primer 338F 0.3 μL, 10 μmol/L primer 806R 0.3 μL, and ddH_2_O up to 10 μL.The amplification procedure consisted of pre-denaturation at 95°C for 3 min, followed by 25 cycles of denaturation at 95°C for 30 s, annealing at 50°C for 30 s, and extension at 72°C for 40 s. Lastly, a final step was performed at 72°C for 7 min. The triplicate products were mixed and electrophoresed in 2% agarose gel at 110 V for 20 min for quality detection (**Supplementary Figure 1**). The PCR recovered products were mixed equally according to their concentration. After clone libraries were constructed using the TruSeq^®^ Nano DNA Kit (Illumina, USA), 2 × 250 bp paired-end high-throughput sequencing was performed using the Illumina NovaSeq platform (Illumina, USA).

### Bioinformatics analysis

Quality control of the sequences was performed through various steps. Firstly, low-quality sequences were removed using Trimmomatic v0.33 software (window size: 50 bp) (12). Primer sequences were then eliminated using Cutadapt v1.9.1 software (Maximum mismatch accepted: 20%; Minimum coverage: 80%) (13). Paired-end reads were assembled using Usearch v10 software (Minimum length of overlap: 10 bp; Minimum similarity within overlapping region: 90%; Maximum mismatch accepted: 5 bp) (14). For OTU clustering and taxonomic annotation of quality control sequences, QIIME 2 software (15) was used to analyze sequences with a similarity of 97% based on the SILVA database (Release132, http://www.arb-silva.de) (16). Alpha diversity indices were calculated using the QIIME 2 software. To assess the species richness of bacterial communities, the ACE index was adopted, with higher values indicating a richer community (17). Furthermore, the Shannon index, which considers both species richness and evenness of bacterial communities, was employed to assess alpha diversity, with higher values indicating greater diversity (18). One-way ANOVA with Tukey’s *post-hoc* test was subsequently employed to analyze the differences in alpha diversity among the three *Rhinopithecus* species. The Principal Coordinates Analysis (PCoA) (19) and Unweighted Pair-group Method with Arithmetic Mean (UPGMA) (20) based on the weighted UniFrac distance were used to analyze the beta diversity. The microbiota differences and similarities between the three *Rhinopithecus* species were analyzed using two statistical methods, specifically Permutational Multivariate Analysis of Variance (PERMANOVA) (21) and Analysis of Similarities (ANOSIM) (22), based on the weighted UniFrac distances in the vegan package of R software. Linear discriminant analysis (LDA) effect size (LEfSe) analysis (23) was used to look for biomarkers that have significant statistical differences among these three species. The Phylogenetic Investigation of Communities by Reconstruction of Unobserved States 2 (PICRUSt 2) software (24) was used to predict the functional gene composition of gut microbiota in the three *Rhinopithecus* species based on the Kyoto Encyclopedia of Genes and Genomes (KEGG) database (25).

## Results

### Sequencing information

**Figure 1:**
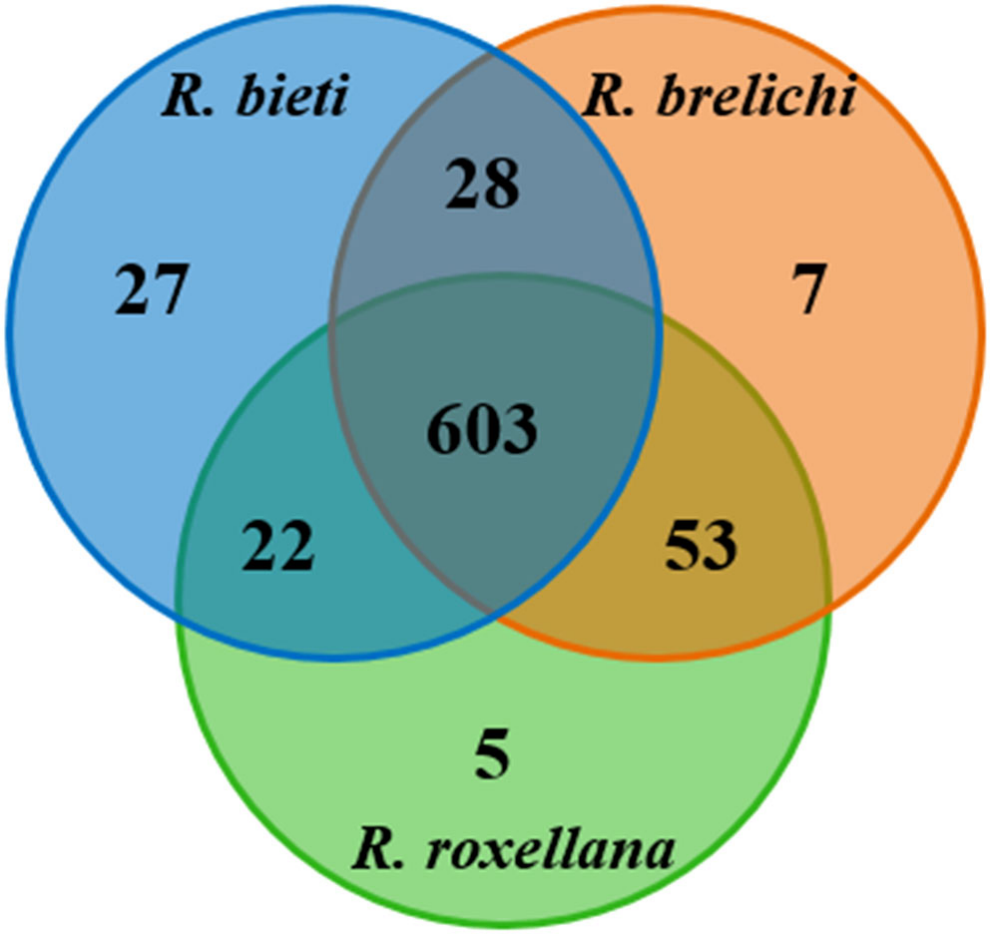
Shared and unique OTUs among the three *Rhinopithecus* species were visualized by Venn diagram.

Of the 1,182,216 effective reads obtained from the samples, 745 OTUs were identified. According to the Venn diagram (**Figure 1**), 603 OTUs were shared among the three *Rhinopithecus* species, largely belonging to Firmicutes (433 OTUs) and Bacteroidetes (61 OTUs). The core bacterial families identified were Ruminococcaceae, Lachnospiraceae, Christensenellaceae, Muribaculaceae, Rikenellaceae, Clostridiales vadinBB60 group, and Bacteroidaceae. Furthermore, *R. bieti*, *R. brelichi*, and *R. roxellana* had 27, 7, and 5 unique OTUs, respectively.

### Analysis of the gut microbiota diversity

**Figure 2:**
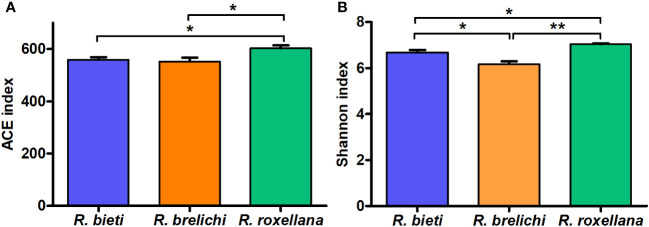
Differences in Alpha diversity of gut microbiota among the three *Rhinopithecus* species. **(A)** Pairwise comparisons of the ACE index between the groups. **(B)** Pairwise comparisons of the Shannon index between the groups. One-way ANOVA with Tukey’s *post-hoc* test: *P < 0.05, **P < 0.01.

**Figure 3:**
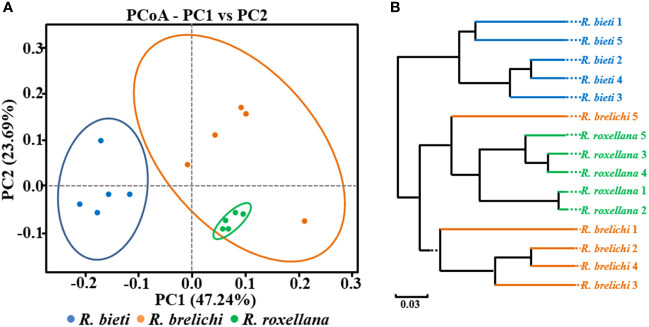
Beta diversity of the gut microbiota in captive snub-nosed monkeys. **(A)** PCoA plot. **(B)** UPGMA clustering tree. Weighted UniFrac algorithm.

As shown in **Figure 2**, the gut microbiota of *R. roxellana* displayed significantly higher ACE and Shannon index values than *R. bieti* and *R. brelichi*, indicating that the gut microbiota of *R. roxellana* was more diverse and richer in microbial taxa. *R. brelichi*, however, displayed the lowest alpha diversity. The weighted UniFrac distance-based PCoA plot in **Figure 3A** showed that the gut microbiota of the *Rhinopithecus* species varied significantly. PERMANOVA analysis (*R. bieti* vs. *R. brelichi*: *R^2^ = *0.487, *P* = 0.007; *R. bieti* vs. *R. roxellana*: *R^2^ = *0.609, *P* = 0.007; *R. brelichi* vs. *R. roxellana*: *R^2^ = *0.320, *P* = 0.016) indicated that the most significant difference in gut microbiota existed between *R. bieti* and *R. roxellana*. In additiona, the UPGMA clustering tree, based on the same distance measure, illustrated a higher similarity in species composition between *R. brelichi* and *R. roxellana* (**Figure 3B**). This similarity was also verified by the ANOSIM analysis (*R. bieti* vs. *R. brelichi*: *R* = 0.788, *P* = 0.007; *R. bieti* vs. *R. roxellana*: *R* = 1.000, *P* = 0.007; *R. brelichi* vs. *R. roxellana*: *R* = 0.480, *P* = 0.027). Comparative genomics assigned *R. brelichi* and *R. roxellana* to northern species and *R. bieti* to Himalayan species (5), suggesting a mutual evolution between the monkeys and their gut microbiota.

### Comparative analysis of the gut microbiota composition

**Figure 4:**
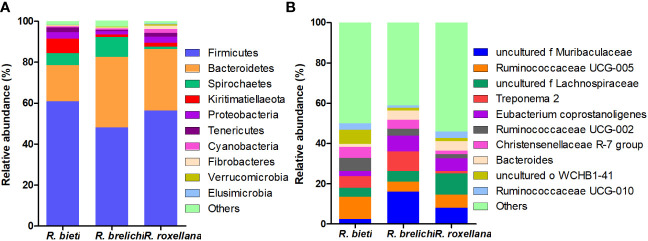
Species distribution of the gut microbiota in captive snub-nosed monkeys. **(A)** The distribution histogram of the top ten phyla in the three groups. **(B)** The distribution histogram of the top ten genera in the three groups.

**Figure 5:**
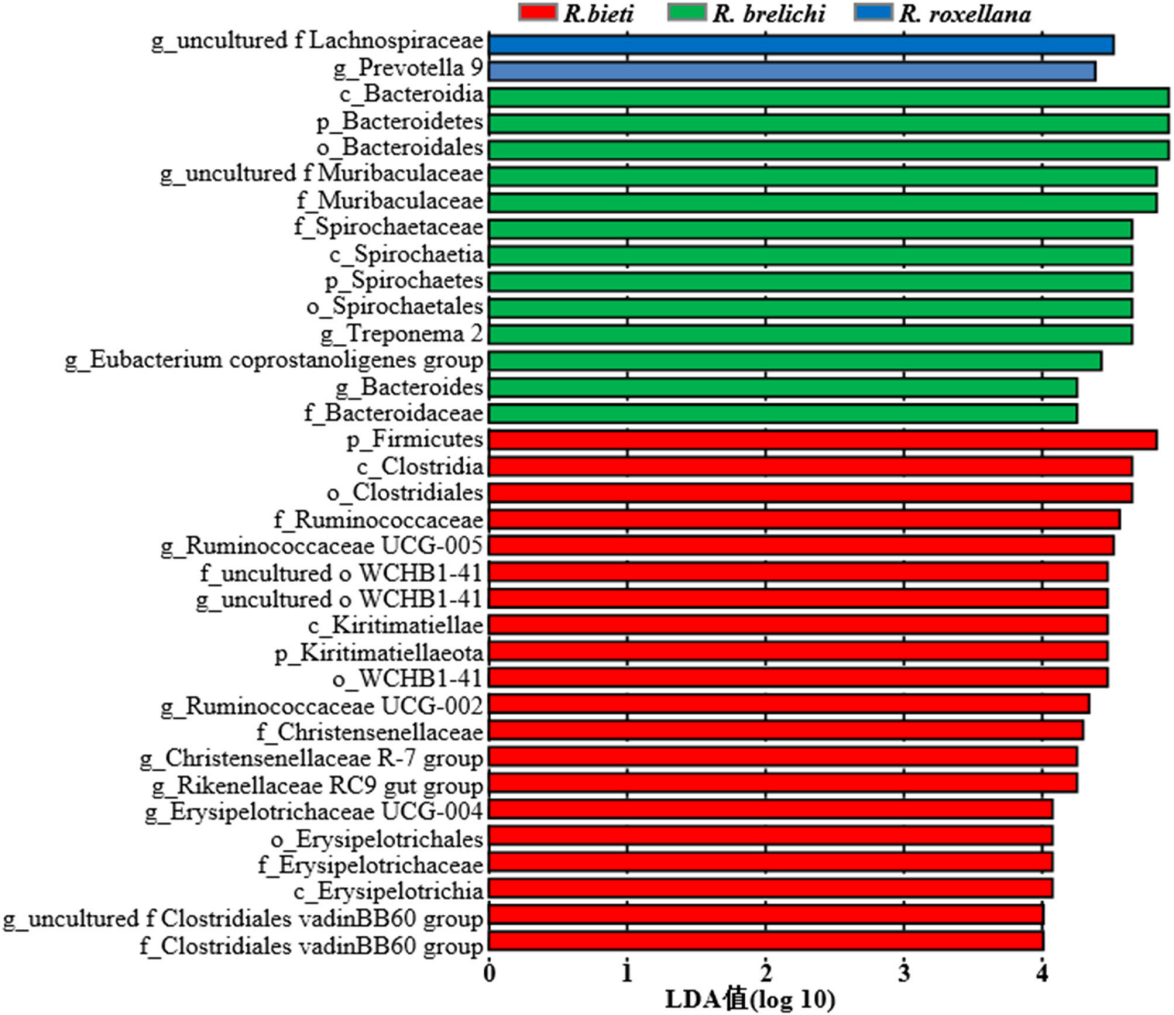
LDA value distribution histogram of the gut microbiota in the captive snub-nosed monkeys. The default LDA threshold is 4.0. Larger LDA values indicate a greater influence of the abundance of bacterial species on the differences in the community between groups.

The top 10 phyla in the gut microbiota of the three *Rhinopithecus* species were shown in **Figure 4A**. The predominant phyla were Firmicutes and Bacteroidetes, accounting for 78.44% in *R. bieti*, 82.56% in *R. brelichi*, and 86.38% in *R. roxellana*. The relative abundance of Firmicutes in the gut of *R. brelichi* was significantly lower than that of *R. bieti* and *R. roxellana* (*P* < 0.01 for *R. brelichi* vs. *R. bieti*; *P* < 0.05 for *R. brelichi* vs. *R. roxellana*), but it had the highest proportion of Bacteroidetes (34.49% for *R. brelichi*; 29.94% for *R. roxellana*; 17.57% for *R. bieti*, *P* < 0.01 for *R. brelichi* vs. *R. bieti*, *P* > 0.05 for *R. brelichi* vs. *R. roxellana*). Among the other nondominant phyla, Kiritimatiellaeota was highest in *R. bieti* (*P* < 0.05 for *R. bieti* vs. *R. brelichi* and *R. bieti* vs. *R. roxellana*), Proteobacteria was lowest in *R. brelichi* (*P* < 0.05 for *R. brelichi* vs. *R. bieti* and *R. brelichi* vs. *R. roxellana*), and Fibrobacteres, Elusimicrobia, and Acidobacteria were highest in *R. roxellana* (*P* < 0.05 for *R. roxellana* vs. *R. bieti* and *R. roxellana* vs. *R. brelichi*) (**Supplementary Table 1**). At the genus level, the gut microbiota of *R. bieti*, *R. brelichi*, and *R. roxellana* were dominated by *Ruminococcaceae UCG-005* (11.06%), *uncultured f Muribaculacea* (16.04%), and *uncultured f Lachnospiraceae* (10.52%), respectively (**Figure 4B**). Out of the top 50 genera, 25 showed significant differences between the three *Rhinopithecus* species (**Supplementary Table 2**). LEfSe analysis showed that the biomarkers of *R. bieti* were Ruminococcaceae (including *Ruminococcaceae UCG-005* and *Ruminococcaceae UCG-002*), Clostridiales vadinBB60 group (*uncultured f Clostridiales vadinBB60 group*), Christensenellaceae (*Christensenellaceae R-7 group*), Erysipelotrichaceae (*Erysipelotrichaceae UCG-004*) belonging to Firmicutes; Kiritimatiellae belonging to Kiritimatiellaeota; uncultured family and genus of WCHB1-41 belonging to Verrucomicrobia; and *Rikenellaceae RC9 gut group* belonging to Bacteroidetes. Meanwhile, Muribaculaceae (*uncultured f Muribaculacea*) and Bacteroidaceae (*Bacteroides*) belonging to Bacteroidetes; Spirochaetaceae (*Treponema 2*) belonging to Spirochaetes; *Eubacterium coprostanoligenes group* belonging to Firmicutes were significantly enriched in *R. brelichi.* The *uncultured f Lachnospiraceae* belonging to Firmicutes and *Prevotella 9* belonging to Bacteroidetes were significantly enriched in *R. roxellana* (**Figure 5**).

### Functional prediction of the gut microbiota

**Figure 6:**
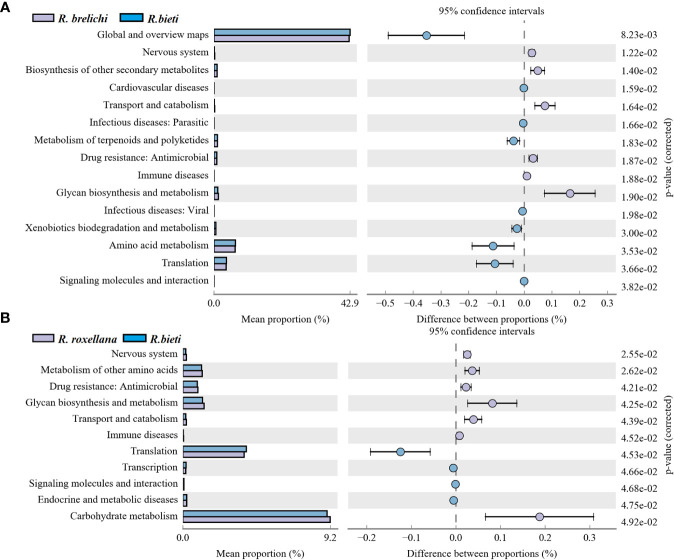
Differences in the KEGG secondary metabolic pathways between the three *Rhinopithecus* species. **(A)** The proportion of different functions between *R. bieti* and *R. brelichi*. **(B)** The abundance proportion of different functions between *R. bieti* and *R. roxellana*. One-way ANOVA (Tukey’s *post-hoc* test), the *P*-value threshold was 0.05.

High-throughput sequencing data of the 16S rRNA V3-V4 gene were analyzed using PICRUSt 2 software based on the KEGG database. The comparison between different *Rhinopithecus* species indicated that the proportion of 15 functional genes varied significantly between *R. bieti* and *R. brelichi* (**Figure 6A**). Specifically, The relative abundance of amino acid metabolism, terpenoids and polyketides metabolism, xenobiotics biodegradation and metabolism, and the global and overview maps pathways of *R. bieti* were significantly higher than that of *R. brelichi.* However, *R. brelichi* showed a significantly higher proportion of the biosynthesis of other secondary metabolites and the glycan biosynthesis and metabolism pathways when compared to *R. bieti*. In addition, within human disease pathways, *R. bieti* showed a higher proportion of parasitic and viral infectious disease, and cardiovascular disease pathways, while *R. brelichi* exhibited a higher proportion of antimicrobial drug resistance, and immune disease pathways. There were 11 functional genes that differed significantly between *R. bieti* and *R. roxellana* (**Figure 6B**). Similar to *R. brelichi*, the glycine biosynthesis and metabolism, antimicrobial drug resistance, and immune diseases were significantly higher in *R. roxellana* than in *R. bieti*. Furthermore, other pathways of amino acid metabolism and carbohydrate metabolism were also significantly higher in *R. roxellana* than in *R. bieti*. No significant difference was observed in carbohydrate metabolism between *R. brelichi* and *R. roxellana*. Overall, *R. bieti* may have different carbohydrate metabolic pathway strategies from the other two *Rhinopithecus* species (**Supplementary Table 3**). There were no significant differences in other secondary and tertiary metabolic pathways between the gut microbiota of *R. brelichi* and *R. roxellana*.

## Discussion

The gut microbiota of the three *Rhinopithecus* species in captive conditions have different characteristics and differences that can be informative for developing captive diet structures, and disease surveillance measures necessary to better protect these endangered species. The study showed that the predominant phyla in snub-nosed monkeys were the Firmicutes and Bacteroidetes, which matches earlier studies (26, 27). Most of the bacterial communities in the Firmicutes can use cellulose, hemicellulose, and xylan as energy sources (28). The relative abundance of Firmicutes in *R. bieti* was significantly higher than in *R. brelichi* and *R. roxellana*, and it was enriched with cellulose or hemicellulose-degrading bacterial families, including Ruminococcaceae (29), Clostridiales vadinBB60 group (30), Christensenellaceae (31) and Erysipelotrichaceae (32). The proportion of the Propanoate and Butanoate metabolism pathway was also significantly higher in *R. bieti*. It is speculated that *R. bieti* may have a stronger ability to decompose plant fibers to produce energy materials. The Bacteroidetes has rich polysaccharide lyase and glycoside hydrolyase genes necessary to break down polysaccharides (33, 34). The Bacteroidetes had the highest abundance in *R. brelichi*, followed by *R. roxellana*, and *R. bieti* showed the lowest percentage. Muribaculaceae and Bacteroidaceae were enriched in *R. brelichi*, and *Prevotella 9* was enriched in *R. roxellana.* Muribaculaceae bacteria are the main users of mucosal sugars (35). Members of Bacteroidaceae provide nutrients to the host by breaking down different glycans (36, 37). The glycan biosynthesis and metabolism pathway was found to be significantly more pronounced in *R. brelichi* and *R. roxellana*, as compared to *R. bieti*. Moreover, these two species demonstrated greater enrichment of important carbohydrate tertiary metabolism pathways such as pentose, glucuronate, starch, and sucrose metabolism, indicating species-specific strategies for carbohydrate metabolism among snub-nosed monkeys.

Apart from the divergence of the predominant phyla, 57.14% of the nondominant phyla showed host differences. Kiritimatiellaeota was found to be the third largest phylum in the gut of *R. bieti* with the highest proportion of genus belonging to order WCHB 1-41. The arginine and fatty acid biosynthesis pathways encoded by the WCHB 1-41 genome were found to play a role in host energy metabolism and nitrogen utilization (38). The comparison of lipid metabolism pathways among the three species of snub-nosed monkeys revealed that the proportion of the fatty acid metabolism in the gut microbiota of *R. bieti* was significantly higher than that in *R. brelichi* and *R. roxellana*. The proportion of Spirochaetes in *R. brelichi* was significantly higher than that in *R. roxellana* (*P* < 0.05). *Treponema 2* was found to be the main genus responsible for this difference (9.69% for *R. brelichi* vs. 1.22% for *R. roxellana, P* < 0.05). *Treponema 2* has enzymes that mediate pyruvate oxidation and decarboxylation to enter the citrate cycle, promoting the biosynthesis of arginine and fatty acids (39). The proportion of Proteobacteria was significantly higher in *R. roxellana*, and the relative abundance of the genus *Parasutterella* was significantly higher than in *R. bieti* and *R. brelichi* (*P* < 0.01). The *Parasutterell* occupied a specific gut niche and could cause changes in bacterial metabolites, such as aromatic amino acids and bile acids in the mouse gut (40). These low-abundance bacterial communities in this study not only caused differences in the composition of the consortium but might also have played a relevant role in interspecies differences in metabolic pathways.

Studies have revealed that northern species (*R. brelichi* and *R. roxellana*) and the Himalayan species (*R. bieti* and *R. strykeri*) diverged about 1.6 million years ago (5). *R. bieti*, which is the largest monkey in the genus *Rhinopithecus*, inhabits virgin alpine forests between 2,500-5,000 meters in southeastern Tibet and northwestern Yunnan (41). *R. strykeri* can only be found in the virgin forests of 1,700-3,100 meters in northwestern Yunnan and northeastern Myanmar (42). *R. brelichi* lives in forests on Fanjing Mountain at an altitude of 500-800 meters in Guizhou Province (43). *R. roxellana* is distributed in forests at an altitude of 1,500-3,300 meters in Sichuan, Gansu, Shaanxi and Hubei provinces of China (44). *R. bieti*, *R. strykeri*, and *R. brelichi* primarily feed on plant such as leaves, shoots, buds, fruits, bark, and lichen (45–47). *R. roxellana* has a more diverse diet and occasionally preys on small animals, birds, eggs, or insects (48). Long-term dietary and habitat differences encourage different hosts to adopt distinct strategies to acquire microbes from nature and attain a healthy and balanced symbiotic relationship (49). This symbiotic relationship makes the microbial communities species-specific and resistant to disturbance over time (50). An analysis of adaptive variations in the gut microbiota of 18 nonhuman primates revealed that the physiological evolution of the host has a greater effect on the construction of the microbiota than the dietary niche (51). However, differences were found when comparing the gut microbiota of wild *R. bieti*, *R. strykeri*, and *R. roxellana* by Wang et al. (2023). Although the gut microbiota of the three *Rhinopithecus* species had similar core bacterial communities involved in degrading cellulose, hemicellulose and lignin, *R. strykeri* had significantly higher levels of enzymes related to pectin and glucose metabolism than *R. bieti* and *R. roxellana*. This difference may be attributed to *R. strykeri*’s greater consumption of fruits, seeds, buds, and flowers, leading to adaptive changes in its gut microbiota. The gut microbiota of *R. bieti* and *R. roxellana*, which share similar lifestyles, were more alike than that of *R. strykeri* (52). Therefore, it can be seen that host species, diet, and habitat have significant effects on the gut microbiota of nonhuman primates. In this study, all snub-nosed monkeys were kept at the Beijing Zoo under the same living environment and dietary structure, which minimized the influence of environmental and dietary factors on the gut microbiota. All samples shared 80.94% of OTUs, mainly annotated to Firmicutes and Bacteroidetes, indicating that snub-nosed monkeys have the same predominant phyla. PCoA and UPGMA clustering analysis showed that *R. brelichi* and *R. roxellana* had a more similar bacterial community composition, and the differential analysis of the KEGG metabolic pathways between these two species did not show significant differences in the metabolic function. However, *R. bieti* (Himalayan species) showed greater differences in both microbial composition and function. Therefore, the profound effects of host species on gut microbiota stability and adaptability must be considered when studying nutritional strategies or the effects of niche on the gut microbiota in snub-nosed monkeys.

## Conclusions

This study investigated the gut microbiota characteristics of three endangered snub-nosed monkey species (*R. bieti*, *R. brelichi*, and *R. roxellana*) in China, under identical captive conditions. Although the predominant phyla of the gut microbiota of the three *Rhinopithecus* species were Firmicutes and Bacteroidetes, the proportions and bacterial communities were found to differ among them. Since most of the bacteria within Firmicutes and Bacteroidetes are involved in carbohydrate metabolism, differences in carbohydrate metabolism pathways were identified between *R. bieti* and the other two *Rhinopithecus* species. Furthermore, 57.14% of the non-dominant phyla were found to differ significantly between the three species. This affected not only microbiota composition but also played a role in the formation of interspecific differences in metabolic function. Finally, *R. brelichi* and *R. roxellana* exhibited greater similarity in gut microbiota composition and metabolic function than *R. bieti*, which is evolutionarily more distant. In conclusion, we obtained valuable insights into the gut microbiota characteristics of endangered snub-nosed monkeys. This study demonstrates the association of host species with gut microbiota, which can be applied to disease surveillance and development of dietary protocols in captive endangered snub-nosed monkeys.

## Data availability statement

The datasets presented in this study can be found in online repositories. The names of the repository/repositories and accession number(s) can be found in the article/**Supplementary Material**.

## Ethics statement

The animal study was approved by Ethics Committee of the Shangqiu Normal University. The study was conducted in accordance with the local legislation and institutional requirements.

## Author contributions

LX designed the experiments and wrote the manuscript. SL and DL collected the samples. JH, SS, and XQ analyzed the data. XW and LZ revised the manuscript. All authors contributed to the article and approved the submitted version.
