# Supplementary material for: Species variations in the gut microbiota of captive snub-nosed monkeys
Source: Front Endocrinol (Lausanne). 2023 Sep 13;14:1250865. doi: 10.3389/fendo.2023.1250865 (PMC10534982; doi:10.3389/fendo.2023.1250865)
Supplement: Supplementary file 1 [file Image_1.pdf]

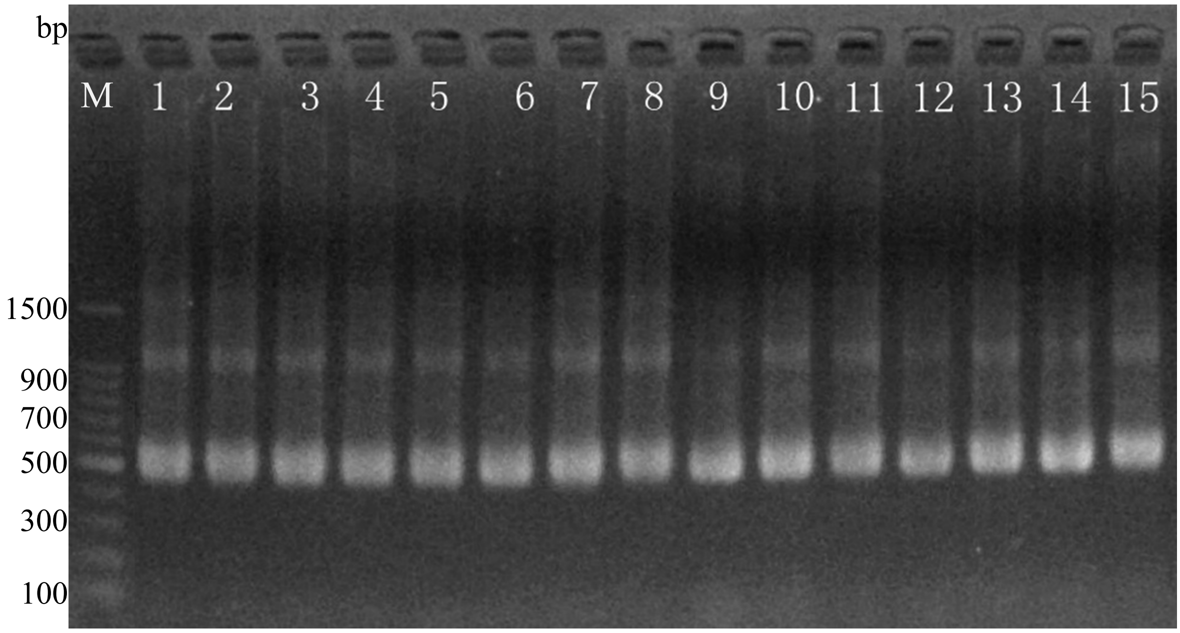

Supplementary Figure 1 Amplification products of the V3-V4 region of the bacterial 16S rRNA gene in captive snub-nosed monkeys. M: 100 bp DNA Ladder (100-1500 bp). Lanes 1-5: Amplification products of *Rhinopithecus bieti*. Lanes 6-10: Amplification products of *R. brelichi*. Lanes 11-15: Amplification products of *R. roxellana.*
