# Supplementary material for: Species variations in the gut microbiota of captive snub-nosed monkeys
Source: Front Endocrinol (Lausanne). 2023 Sep 13;14:1250865. doi: 10.3389/fendo.2023.1250865 (PMC10534982; doi:10.3389/fendo.2023.1250865)
Supplement: Supplementary file 2 [file Table_1.docx]

Supplementary Table S1 Differences in relative abundance of phyla between the three *Rhinopithecus* species (One-way ANOVA with Tukey’s post-hoc test).

| Phylum | *R. bieti*  (%) | *R. brelichi*  (%) | *R. roxellana*  (%) | *R. bieti*  vs  *R. brelichi*  (*P*) | *R. bieti*  vs  *R. roxellana*  (*P*) | *R. brelichi*  vs  *R. roxellana*  (*P*) |
| --- | --- | --- | --- | --- | --- | --- |
| Firmicutes | 60.87 | 48.08 | 56.43 | **0.002** | 0.289 | **0.028** |
| Bacteroidetes | 17.57 | 34.49 | 29.94 | **0.000** | **0.005** | 0.346 |
| Spirochaetes | 5.94 | 9.70 | 1.23 | 0.369 | 0.223 | **0.021** |
| Kiritimatiellaeota | 6.97 | 1.17 | 1.72 | **0.013** | **0.023** | 0.945 |
| Proteobacteria | 3.35 | 1.67 | 3.05 | **0.013** | 0.819 | **0.039** |
| Tenericutes | 2.27 | 0.72 | 1.92 | **0.016** | 0.743 | 0.060 |
| Cyanobacteria | 0.43 | 0.62 | 1.83 | 0.918 | **0.030** | 0.059 |
| Fibrobacteres | 0.50 | 0.50 | 1.62 | 1.000 | **0.002** | **0.002** |
| Verrucomicrobia | 0.21 | 0.36 | 0.70 | 0.765 | 0.086 | 0.264 |
| Elusimicrobia | 0.10 | 0.02 | 0.31 | 0.442 | **0.016** | **0.002** |
| Actinobacteria | 0.14 | 0.11 | 0.17 | 0.820 | 0.801 | 0.451 |
| Epsilonbacteraeota | 0.13 | 0.05 | 0.06 | 0.062 | 0.087 | 0.978 |
| Lentisphaerae | 0.09 | 0.11 | 0.01 | 0.959 | 0.346 | 0.235 |
| Fusobacteria | 0.02 | 0.02 | 0.01 | 0.632 | 0.141 | 0.518 |
| Acidobacteria | 0.00 | 0.00 | 0.02 | 0.994 | **0.037** | **0.045** |
| Patescibacteria | 0.01 | 0.01 | 0.00 | 0.194 | 0.079 | 0.849 |
