# Supplementary material for: Species variations in the gut microbiota of captive snub-nosed monkeys
Source: Front Endocrinol (Lausanne). 2023 Sep 13;14:1250865. doi: 10.3389/fendo.2023.1250865 (PMC10534982; doi:10.3389/fendo.2023.1250865)
Supplement: Supplementary file 3 [file Table_2.docx]

Supplementary Table S2 Differences in relative abundance of genera (top 50) between the three *Rhinopithecus* species (One-way ANOVA with Tukey’s post-hoc test).

| Genus | *R. bieti*  (%) | *R. brelichi*  (%) | *R. roxellana*  (%) | *R. bieti*  vs  *R. brelichi*  (*P*) | *R. bieti*  vs  *R. roxellana*  (*P*) | *R. brelichi*  vs  *R. roxellana*  (*P*) |
| --- | --- | --- | --- | --- | --- | --- |
| uncultured f Muribaculaceae | 2.49 | 16.04 | 8.14 | **0.002** | 0.176 | **0.048** |
| Ruminococcaceae UCG-005 | 11.06 | 4.98 | 6.48 | **0.008** | **0.041** | 0.643 |
| uncultured f Lachnospiraceae | 4.34 | 5.29 | 10.52 | 0.816 | **0.005** | **0.014** |
| Treponema 2 | 5.92 | 9.69 | 1.22 | 0.367 | 0.225 | **0.021** |
| Eubacterium coprostanoligenes | 2.42 | 7.95 | 6.23 | **0.000** | **0.001** | 0.103 |
| Ruminococcaceae UCG-002 | 6.54 | 3.29 | 2.01 | 0.055 | **0.009** | 0.576 |
| Christensenellaceae R-7 group | 5.46 | 4.48 | 1.78 | 0.625 | **0.010** | 0.056 |
| Bacteroides | 1.61 | 4.71 | 4.67 | 0.050 | 0.054 | 0.999 |
| uncultured o WCHB1-41 | 6.97 | 1.17 | 1.72 | **0.013** | **0.023** | 0.945 |
| Ruminococcaceae UCG-010 | 3.21 | 1.26 | 3.15 | 0.091 | 0.997 | 0.103 |
| Ruminococcus 1 | 3.23 | 1.60 | 2.52 | **0.022** | 0.393 | 0.221 |
| Rikenellaceae RC9 gut group | 4.46 | 0.81 | 1.87 | **0.001** | **0.012** | 0.360 |
| uncultured bacterium o Bacteroidales | 2.05 | 3.71 | 0.75 | 0.402 | 0.566 | 0.082 |
| Prevotella 9 | 0.01 | 1.32 | 5.06 | 0.706 | **0.022** | 0.092 |
| uncultured bacterium f Prevotellaceae | 1.65 | 2.65 | 1.68 | 0.845 | 1.000 | 0.852 |
| Ruminococcaceae NK4A214 group | 2.34 | 1.73 | 1.59 | 0.569 | 0.431 | 0.968 |
| uncultured bacterium f Ruminococcaceae | 1.51 | 1.59 | 2.35 | 0.978 | 0.101 | 0.142 |
| Alistipes | 0.89 | 2.35 | 2.10 | **0.041** | 0.094 | 0.880 |
| Phascolarctobacterium | 2.31 | 1.01 | 1.42 | 0.050 | 0.202 | 0.686 |
| uncultured bacterium k Bacteria | 1.39 | 2.38 | 0.97 | 0.653 | 0.923 | 0.432 |
| Ruminococcaceae UCG-014 | 1.52 | 1.35 | 1.59 | 0.956 | 0.993 | 0.917 |
| Parabacteroides | 0.80 | 0.54 | 2.99 | 0.944 | **0.047** | **0.026** |
| Faecalibacterium | 0.28 | 1.98 | 1.45 | **0.021** | 0.120 | 0.598 |
| uncultured bacterium f Clostridiales vadinBB60 group | 2.53 | 0.36 | 0.79 | **0.045** | 0.113 | 0.852 |
| Ruminococcaceae UCG-013 | 0.40 | 0.69 | 2.21 | 0.908 | 0.057 | 0.117 |
| uncultured bacterium o Mollicutes RF39 | 1.72 | 0.72 | 0.86 | **0.013** | **0.033** | 0.869 |
| uncultured bacterium o Rhodospirillales | 0.67 | 0.80 | 1.73 | 0.944 | 0.057 | 0.099 |
| uncultured bacterium f Christensenellaceae | 1.24 | 1.19 | 0.59 | 0.995 | 0.451 | 0.505 |
| Prevotellaceae UCG-001 | 0.78 | 1.39 | 0.74 | 0.141 | 0.991 | 0.114 |
| Erysipelotrichaceae UCG-004 | 2.58 | 0.13 | 0.06 | **0.029** | **0.025** | 0.996 |
| Fibrobacter | 0.50 | 0.50 | 1.62 | 1.000 | **0.002** | **0.002** |
| uncultured bacterium o Gastranaerophilales | 0.40 | 0.45 | 1.75 | 0.994 | **0.028** | **0.034** |
| uncultured bacterium o Clostridiales | 1.20 | 0.68 | 0.39 | 0.258 | 0.056 | 0.628 |
| Roseburia | 0.58 | 0.41 | 1.06 | 0.909 | 0.471 | 0.273 |
| Ruminococcaceae UCG-009 | 0.87 | 0.70 | 0.46 | 0.602 | 0.094 | 0.410 |
| Lachnospira | 0.23 | 0.26 | 1.46 | 0.997 | **0.006** | **0.007** |
| Butyrivibrio | 1.04 | 0.88 | 0.02 | 0.978 | 0.456 | 0.569 |
| Anaerovibrio | 0.69 | 0.48 | 0.66 | 0.756 | 0.994 | 0.816 |
| uncultured bacterium o Bradymonadales | 1.70 | 0.00 | 0.01 | **0.028** | **0.028** | 1.000 |
| Family XIII AD3011 group | 0.34 | 0.36 | 0.91 | 0.997 | 0.142 | 0.160 |
| Prevotellaceae UCG-003 | 0.36 | 0.18 | 1.02 | 0.854 | 0.192 | 0.080 |
| Oscillibacter | 0.57 | 0.42 | 0.56 | 0.402 | 0.998 | 0.428 |
| Ruminiclostridium 6 | 0.55 | 0.19 | 0.70 | 0.551 | 0.889 | 0.312 |
| uncultured bacterium f Bacteroidales RF16 group | 1.31 | 0.09 | 0.00 | **0.000** | **0.000** | 0.872 |
| Prevotella 7 | 0.44 | 0.35 | 0.35 | 0.933 | 0.925 | 1.000 |
| uncultured bacterium o Izimaplasmatales | 0.03 | 0.01 | 1.06 | 0.997 | **0.015** | **0.013** |
| Ruminococcus 2 | 0.76 | 0.15 | 0.17 | 0.187 | 0.199 | 0.999 |
| Parasutterella | 0.17 | 0.19 | 0.66 | 0.986 | **0.004** | **0.006** |
| Cerasicoccus | 0.00 | 0.34 | 0.60 | 0.189 | **0.018** | 0.386 |
| Marvinbryantia | 0.34 | 0.20 | 0.39 | 0.285 | 0.831 | 0.116 |
