# Supplementary material for: Species variations in the gut microbiota of captive snub-nosed monkeys
Source: Front Endocrinol (Lausanne). 2023 Sep 13;14:1250865. doi: 10.3389/fendo.2023.1250865 (PMC10534982; doi:10.3389/fendo.2023.1250865)
Supplement: Supplementary file 4 [file Table_3.docx]

Supplementary Table S3 Differences in Carbohydrate tertiary metabolic pathways between the gut microbiota of the three *Rhinopithecus* species (One-way ANOVA with Tukey’s post-hoc test).

| Metabolic pathway | *R. bieti*  (%) | *R. brelichi*  (%) | *R. roxellana*  (%) | *R. bieti*  vs  *R. brelichi*  (*P*) | *R. bieti*  vs  *R. roxellana*  (*P*) | *R. brelichi*  vs  *R. roxellana*  (*P*) |
| --- | --- | --- | --- | --- | --- | --- |
| Amino sugar and nucleotide sugar metabolism | 1.07 | 1.09 | 1.11 | 0.370 | **0.049** | 0.387 |
| Ascorbate and aldarate metabolism | 0.05 | 0.06 | 0.06 | 0.259 | **0.039** | 0.347 |
| Butanoate metabolism | 0.62 | 0.58 | 0.57 | **0.049** | **0.038** | 0.363 |
| C5-Branched dibasic acid metabolism | 0.27 | 0.26 | 0.26 | 0.480 | 0.095 | 0.917 |
| Citrate cycle (TCA cycle) | 0.62 | 0.60 | 0.58 | 0.417 | 0.124 | 0.101 |
| Fructose and mannose metabolism | 0.60 | 0.63 | 0.64 | 0.484 | 0.111 | 0.633 |
| Galactose metabolism | 0.49 | 0.62 | 0.61 | **0.022** | **0.018** | 0.775 |
| Glycolysis / Gluconeogenesis | 1.03 | 1.04 | 1.04 | 0.805 | 0.610 | 0.906 |
| Glyoxylate and dicarboxylate metabolism | 0.67 | 0.69 | 0.68 | 0.053 | **0.039** | 0.379 |
| Inositol phosphate metabolism | 0.12 | 0.12 | 0.12 | 0.565 | 0.960 | 0.514 |
| Pentose and glucuronate interconversions | 0.29 | 0.31 | 0.32 | 0.203 | **0.013** | 0.523 |
| Pentose phosphate pathway | 0.72 | 0.73 | 0.76 | 0.471 | 0.052 | 0.229 |
| Propanoate metabolism | 0.59 | 0.53 | 0.54 | **0.018** | **0.033** | 0.479 |
| Pyruvate metabolism | 1.11 | 1.08 | 1.08 | 0.263 | 0.076 | 0.836 |
| Starch and sucrose metabolism | 0.74 | 0.81 | 0.81 | 0.082 | **0.023** | 0.817 |
